# Novel Dihydroxy-Containing Ammonium Phosphate Based Poly(Lactic Acid): Synthesis, Characterization and Flame Retardancy

**DOI:** 10.3390/polym10080871

**Published:** 2018-08-05

**Authors:** Rong-Kun Jian, Long Xia, Yuan-Fang Ai, De-Yi Wang

**Affiliations:** 1 Fujian Provincial Key Laboratory of Polymer Materials, College of Chemistry and Materials Science, Fujian Normal University, Fuzhou 350007, China; jrkht1987@fjnu.edu.cn (R.-K.J.); xialong2018@163.com (L.X.); aiyf114@163.com (Y.-F.A.); 2 IMDEA Materials Institute, C/Eric Kandel, 2, 28906 Getafe, Spain

**Keywords:** poly(lactic acid), flame retardancy, thermal stability, mechanism

## Abstract

The aim of this work is to prepare flame-retardant biobased poly(lactic acid) materials through incorporating a novel flame retardant dihydroxy-containing ammonium phosphate (DAP) derived from 2-chloro-5,5-dimethyl-1,3,2-dioxaphosphinane-2-oxide (DOP) and 2-amino-2-methyl-1,3-propanediol (AMPD). Interestingly, PLA modified with only 0.5% DAP passed UL-94 V-0 rating, and possessed a limiting oxygen index (LOI) value of 24.6%, which would further increase with the increasing loading of DAP. PLA/DAP did not exhibit obviously improved results in terms of heat release rate (HRR), as the loading of DAP was relatively low. It was found that DAP showed little effect on the thermal stability of PLA and the onset decomposition temperatures of PLA and PLA/DAP blends were very close. Besides, the degree of crystallization increased because of the plasticized effect of DAP. Based on the analyses of flame-retardant mechanism of DAP, it disclosed that DAP decomposed to generate incombustible compounds, such as water and ammonia, to dilute the concentration of oxygen and fuels, and then release some phosphorus-containing fragments that could produce phosphorus-containing free radicals to interrupt free-radical reactions, and finally noncombustible melt dripping was produced so as to bring away large amount of heat and stop the feedback of heat to the matrix.

## 1. Introduction

Nowadays, concerns over the overuse of non-renewable petroleum-based polymers and inevitable accumulation of non-degradable plastic waste have propelled people to develop eco-friendly polymers from renewable resources, such as crops and plants [1,2]. Poly(lactic acid) (PLA), as a biobased polymer derived from corn and starch, now has wide application in packaging, agricultural film, biomedical, and pharmaceutical fields [3,4,5,6]. However, some typical problems, such as the flammability, serious melt-drip during combustion, and slow crystallization, have also restricted, to some extent, the industrial application of PLA [7,8]. In this case, PLA should be modified to meet the requirement of industrial application.

Currently, the major strategies to improve the flame retardancy of PLA include chemical modification and physical blending with flame retardants (FRs) [9,10,11,12]. Therein, the chemical methods, such as copolymerization and grafting with monomers containing flame-retardant elements, can improve the flame retardancy of PLA and compatibility with hydrophilic polymers [13,14,15]. However, the drawbacks are put forward that the degree of crystallization and the glass transition temperature (*T*_g_) of PLA undergo a remarkable decrease [14]. By contrast, the physical method is a direct and effective way to simultaneously reduce the flammability of polymers and maintain thermal properties [16,17,18]. The commonly reported flame retardants can be classified into nanoparticles [19,20,21], phosphorus-based [17,18,22,23], and intumescent flame retardants (IFRs) [24,25,26,27]. Among them, phosphorus-based FRs and IFRs are well developed recently, thanks to their high flame-retardant efficiency. In details, it was reported by Liu et al. that the combination of ammonium polyphosphate and synthesized charring agent with the loading of 24 wt % and 6 wt %, respectively, imparted PLA with good flame retardancy [28]. Besides, Long et al. designed three bis phosphorus-carbon bridged 9,10-dihydro-9-oxa-10-phosphaphe nanthrene-10-oxide (DOPO) derivatives, and incorporated them into PLA. It was found that with 10 wt % addition of these derivatives, all PLA composites achieved a UL-94 V-0 rating, and the LOI value of PLA/DiDOPO1 increased from 20.0% of PLA to 36.4% [29]. However, it is seen that to acquire satisfactory results, the high loading of these additives (more than 10 wt %) is needed, which in turn deteriorates other properties of PLA, such as thermal stabilities. To date, some novel ammonium phosphates have been reported to exert high flame-retardant effect on polymers. For example, Li et al. developed a biobased ammonium phytate (PA-HDA) to react, in situ, with isocynate (MDI) during the melt-blending process, so as to simultaneously toughen and flame-retard PLA. The result showed that with only 5 wt % PA-HDA loading, PLA passed UL-94 V-0 rating, and its limiting oxygen index (LOI) value was as high as 26% [11]. Inspired from the phenomenon, a novel organic ammonium phosphate (DAP) was synthesized with DOP and AMPD in consideration of the high flame-retardant efficiency of DOP and the multifunctional groups of AMPD that will help the target compound, DAP, successfully be synthesized.

This work was aimed at reducing the inflammability of PLA and maintaining the thermal stabilities of PLA. First of all, through a one-pot reaction, the targeted flame-retardant DAP was synthesized using DOP and AMPD as the reactants. Afterwards, the target was well characterized, and its chemical structure was confirmed. The flame-retardant PLA materials were then prepared by melt-blending with the quantitative DAP through a torque rheometer. Besides, the thermal stability, flame retardancy, and the relevant flame-retardant mechanism of the flame-retardant PLA were all well investigated, respectively.

## 2. Experimental Section 

### 2.1. Materials

Poly(lactic acid) (4032D, Nature Works, Blair, NE, USA) was commercially obtained. 2-amino-2-methyl-1,3-propanediol was purchased from Macklin Biochemical Co., Ltd. (Shanghai, China) Phosphoryl trichloride and dimethyl-1,3-propanediol were both bought from Xiya Chemical industries Co., Ltd. (Chengdu, China). The reagents acetone, triethylamine (TEA), and chloroform without purification, were directly supplied by Zhiyuan Chemical Reagent Co., Ltd. (Tianjin, China).

### 2.2. Synthesis of DAP

**Scheme 1:**
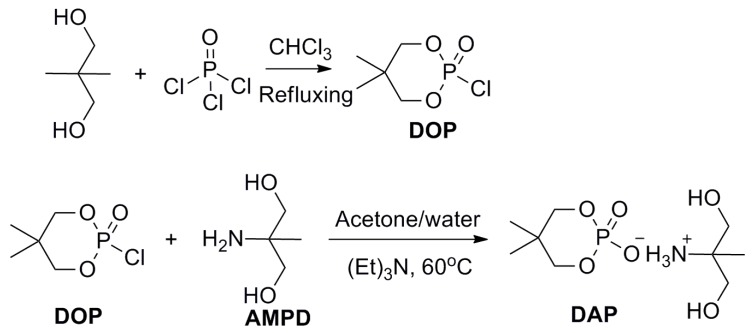
The synthetic route of dihydroxy-containing ammonium phosphate (DAP).

DOP was synthesized according to the previously reported literature [30]. The detailed synthetic process of DAP was as follows: 18.5 g DOP (0.1 mol) dispersed in 100 mL solution of acetone/water (volume ratio, 1:2) was transferred into a 500 mL three-neck round flask and kept at room temperature. Afterwards, the solution of 10.2 g AMPD (0.1 mol) and 28 mL TEA dissolved in 100 mL solution of acetone/water was slowly added to the flask, and the reaction system became gradually transparent. Thereafter, the reaction system was heated to 60 °C, and continued to react for 8 h. Finally, the crude product was obtained through removing the solvent with a rotary evaporator, and washed with trichloromethane three times, and then dried in vacuum at 60 °C for 8 h to obtain a white compound with a yield of 70 wt %. The synthetic route is shown in Scheme 1. 

### 2.3. Preparation of PLA/DAP

PLA was dried in vacuum at 80 °C for 8 h prior to use. PLA/DAP blends were prepared using a torque rheometer (Haake, Polylab QC, Karlsruhe, Germany). PLA was first added into the chamber of the rheometer at 185 °C and 50 rpm for 4 min, and then DAP was incorporated to PLA and mixed for 4 min. Additionally, neat PLA was also processed according to the aforementioned procedure as the control. PLA blends with 0.25 wt %, 0.5 wt %, 1 wt %, and 2 wt % DAP were coded as PLA1, PLA2, PLA3, and PLA4 as shown in Table 1, respectively. Finally, these PLA blends were shattered into small particles, and then compression-molded in 10 MPa at 185 °C.

### 2.4. Methods

Fourier-transform infrared spectroscopy (FTIR) spectra of specimens (KBr pellets) were recorded on a Thermo Nicolet 5700 FTIR instrument (Thermo Nicolet Co., Madison, WI, USA) in the range of 4000–450 cm^−1^. 

Nuclear magnetic resonance (NMR) tests in terms of ^1^H NMR and ^31^P NMR were conducted on a Bruker AVANCE AV II-400 NMR instrument (Bruker Co., Billerica, MA, USA) using DMSO-*d*_6_ as the solvent.

Differential scanning calorimetry (DSC) tests were conducted through Mettler-Toledo DSC822e instrument (Mettler Toledo, Greifensee, Switzerland). The specified testing condition was set as follows: in the nitrogen atmosphere, the material was firstly treated in the range of 30 to 200 °C with a temperature-increasing rate of 10 °C min^−1^, and maintained at 200 °C for five minutes to remove the heat history. Thereafter, it was gradually cooled down below 30 °C, and then re-heated with the same procedure as the former. Based on the derivative thermal parameters listed below, the crystallinity (*X_c_*) of PLA-based materials were calculated according to Equation (1):

(1) $$\chi_{c}=\frac{\Delta H_{m}-\Delta H_{c}}{w_{\mathrm{PLA}}\times \Delta H_{m}^{0}}\times 100,$$

where $\Delta H_{c}$ is the crystallization enthalpy (J·g^−1^), $\Delta H_{m}$ is the enthalpy of melting, and $\Delta H_{m}^{0}$ is the melting enthalpy for the 100% crystalline PLA equal to 93 J·g^−1^, and $w_{\mathrm{PLA}}$ is the weight fraction of PLA. 

The thermal stabilities of samples were assessed through thermogravimetric analyses (TG) which were carried out on a METTLER TGA/SDTA 851 thermal analyzer (Mettler Toledo, Greifensee, Switzerland) from 30 to 600 °C at a heating rate of 10 °C min^−1^ under nitrogen atmosphere.

The gaseous products of samples from the heating treatment of TG were analyzed through FTIR linked to the parts of TG apparatus with a heated transfer line set at 280 °C. The heat-treatment procedure was set from 40 to 700 °C at a heating rate of 10 °C min^−1^ under the nitrogen atmosphere at a purge flow rate of 60 mL min^−1^.

Specimens with the three-dimensional size of 130.0 × 6.5 × 3.2 mm^3^ were experimented on a HC-2C oxygen index meter ((Jiangning Analytical Instrument Co., Jiangning, China) to collect the limiting oxygen index (LOI) according to ASTM D2863-97. Samples with the three-dimensional size of 130.0 × 13 × 3.2 mm^3^ were conducted on a CZF-2 instrument ((Jiangning Analytical Instrument Co., Jiangning, China) to obtain the UL-94 V rating according to ASTM D3801-2010. In addition, fire behaviors of samples with three-dimensional size of 100.0 × 100.0 × 3.0 mm^3^ were assessed through an FTT M1354 cone calorimeter device (Fire Testing Technology Ltd., East Grinstead, UK) according to ISO 5660-1, and the samples were exposed to a radiant cone at a heat flux of 35 kW m^−2^.

Flame retardant was pyrolyzed at 500 °C in the CDS5200 pyrolyzer linked to the chromatograph/mass spectrometer (py-GC/MS, Clarus 680 GC-SQ8MS, Perkin-Elmer, Inc., Waltham, MA, USA). The gas-phase products were then carried by helium to the MS indicator at 280 °C. For the part of MS indicator, it was monitored under the electron impact mode at 70 eV with the ion source temperature of 250 °C. Finally, the mass spectra of products were first identified according to NIST MS library, and then processed with Origin 8. 

## 3. Results and Discussion

### 3.1. Synthesis and Characterization of DAP

**Scheme 2:**
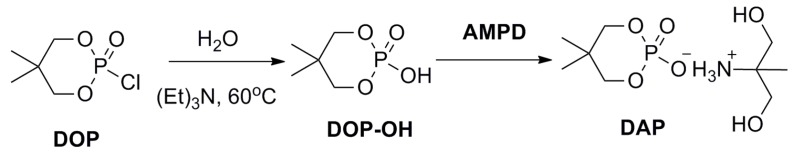
The proposal synthetic process of DAP.

Although DAP was synthesized with a one-pot method, the formation of DAP experienced two steps as shown in Scheme 2. Normally, DOP firstly experienced hydrolysis to form an intermediate DOP–OH, and the intermediate then reacted with AMPD to produce the target through an acid–base neutralization reaction. 

**Figure 1:**
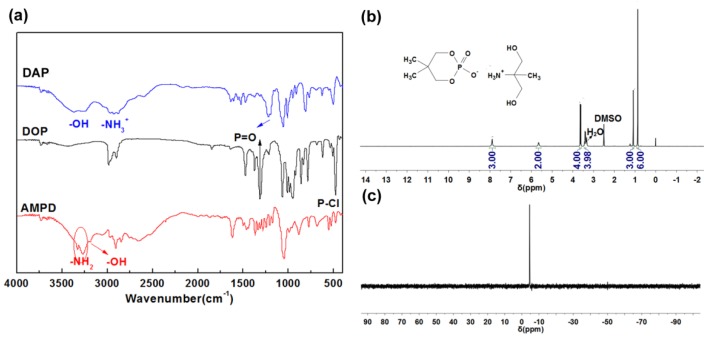
FTIR (**a**), ^1^H NMR (**b**), and ^31^P NMR (**c**) spectra of DAP.

The chemical structure of DAP is confirmed by FTIR, ^1^H NMR, and ^31^P NMR, as seen in Figure 1, respectively. From the spectrum of DAP, it can be found that the peak ascribed to P–Cl at 540 cm^−1^ is not detected, as well as the double absorbing peaks corresponding to primary amino (–NH_2_) of AMPD at 3332 and 3257 cm^−1^. On the contrary, a broad absorbing band at about 3100 and 2500 cm^−1^ assigned to –NH_3_^+^ appears. Additionally, the signal of P=O is also found at 1212 cm^−1^. From the ^1^H NMR spectrum, it is observed that the single peak at 7.89 ppm is ascribed to the hydrogen of –NH_3_^+^, and the signal located at 5.65 ppm belongs to –OH. Moreover, the double signals at 3.63 ppm corresponded to –CH_2_–O– in the ring structure, and the multi-peaks of –CH_2_–OH are found at 3.40 ppm. The two single peaks at 1.09 and 0.87 ppm are ascribed to –CH_3_. It is also noted that the calculated number of hydrogens matches well with the theoretical. In addition, only one signal is found in the ^31^P NMR spectrum, indicating DAP has high purity. Based on the above analyses, it is confirmed that the target flame retardant has been successfully synthesized.

### 3.2. Burning Behaviors 

PLA and PLA/DAP blends were tested in terms of LOI tests and UL-94 vertical burning tests to assess their flame retardancy, and the results are listed in Table 1. As discussed previously, PLA is actually an inflammable polymer, and its LOI value is only 19.5% lower than the oxygen concentration in the atmosphere, indicating PLA is easily ignited once it contacts with fire. Besides, during combustion, PLA generates severe melt drippings which ignite the cotton, so that it cannot pass the UL-94 rating. In the case of PLA/DAP blends, LOI values are markedly increased to 23.0%, 24.6%, 30.3%, and 38.3%, respectively, for PLA1, PLA2, PLA3, and PLA4. It is known that the higher the LOI value, the better the ignition resistance. While, as the loading of DAP in PLA is so low that the melt drippings still ignite the cotton, and PLA1 fails to pass UL-94 rating. As for PLA2, PLA3, and PLA4, they are hard to ignite, and have quick self-extinguishing ability after being removed from the igniter. It should be noted that the produced melt drippings cannot ignite the cotton. Therefore, PLA2, PLA3, and PLA4 all pass UL-94 V-0 ratings. According to the analyses above, it indicates that DAP shows a high efficiency on flame-retarding PLA.

**Figure 2:**
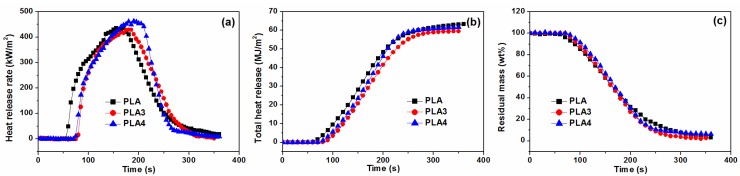
The curves of heat release rate (**a**), total heat release (**b**), and residue mass (**c**) versus time for PLA and PLA/DAP blends.

Besides, cone calorimeter (CC) tests, as a classical fire testing technique that simulates real-scale fire conditions [31,32], were conducted to assess the combustion behaviors of PLA, PLA3, and PLA4, respectively. The detailed results and corresponding data, such as time to ignition (TTI), peak heat release rate (PHRR), time to PHRR (TTP), and total heat release (THR), average effective heat of combustion (av-EHC), and total smoke production (TSP), are summarized in Figure 2 and Table 2. 

TTI is always used to evaluate the ignition resistance of materials, and a long TTI value indicates that the material is difficult to ignite [32]. It is seen that PLA has a TTI value of 54 s. By contrast, the TTI values of the PLA3 and PLA4 are 71 and 69 s, respectively. That means that PLA/DAP blends are difficult to be ignited when exposed to the heat flux of 35 kW m^−2^. The lower decomposition temperature of DAP might be the key to the improvement of the ignition resistance of PLA, as its decomposition products have some activities in flame suppression. On the other hand, the decreased molecular weight of PLA induced by the decomposition products of DAP also causes PLA to rapidly melt, leading to enhance the ignition resistance.

After ignition, the HRR value of PLA rapidly increases to the peak value of 436 kW m^−2^ at 170 s. As for PLA/DAPs, it is odd that the PHRR values have no reduction as compared to that of PLA. Nevertheless, it should be mentioned that the fire growth rate (FIGRA), equal to the ratio of PHRR to TTP, is considered as an important parameter for the estimation of the fire-spreading rate and fire risk [33]. Besides, a lower FIGRA value means a slower flame-spread rate and longer time for people to escape from a fire. By comparison, the FIGRA values of PLA/DAP are lower than that of PLA, demonstrating that PLA/DAP represents a lower fire hazard. 

**Figure 3:**
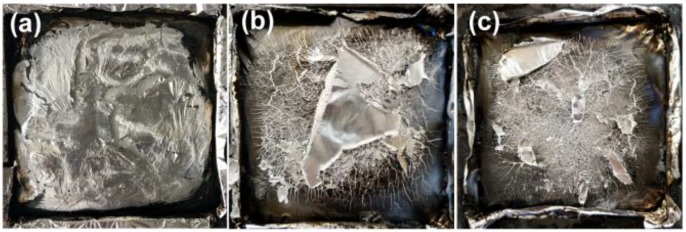
Digital photos of residues after cone calorimeter tests for PLA (**a**), PLA3 (**b**), and PLA4 (**c**).

After complete combustion, the THR values of PLA/DAP are also lower than that of PLA. Moreover, it seems that there is little improvement on the char residues when PLA is modified with DAP, because the morphologies as seen in Figure 3 show that there is little char left, and the curves of residual mass versus time also prove this. Moreover, it is also found that the TSP values of PLA3 and PLA4 are similar to that of PLA, and the av-EHC values of PLA3 and PLA4 are both lower than that of neat PLA, indicating that DAP acted in the gaseous phase. These phenomena suggest that DAP might exert minor flame-retarding effect in the char formation.

Consequently, based on the results of LOI, UL-94 rating and CC tests, it strongly reveals that the PLA/DAP blends have a rather low flammability and improved ignition resistance under the action of DAP combining with vapor phase flame inhibition and promotion of melting process.

### 3.3. Thermal Analysis

**Figure 4:**
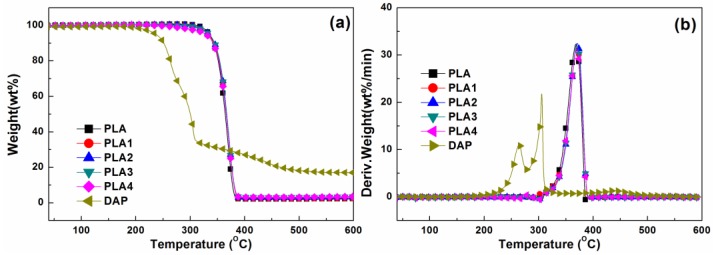
TG (**a**) and DTG (**b**) curves of PLA and PLA/DAP under nitrogen atmosphere.

Thermal stabilities of all the materials were tested and compared, and the TG and DTG curves are given in Figure 4, whereas Table 3 shows the corresponding data. It is observed that DAP is stable up to about 237 °C, equal to the temperature at 5 wt % weight loss, and later gets to the maximum weight loss rate of 21.7 wt % min^−1^ at 306 °C (*T*_max_), and finally, the residue of 17.1 wt % is left at 600 °C. As for PLA, *T*_5%_ displayed at 335 °C is higher than that of DAP, and then accompanies with a maximum mass loss rate of 32.5 wt % min^−1^ at 368 °C. As a consequence, almost no residue is left. It seems that the addition of DAP would cause the decreased thermal stability of PLA. In fact, *T*_5%_ values, *T*_max_ values, and the maximum mass loss rates of PLA/DAP blends have little change as compared to PLA. Besides, it is observed that char residues of PLA1, PLA2, PLA3, and PLA4 only show a slight improvement, meaning that DAP cannot exert strong influence on char-formation of PLA. It is speculated that the low addition of DAP is the explanation to this phenomenon, and it does not have enough ability to affect the thermal stability of PLA. Generally, DAP does not obviously influence the thermal stability of PLA.

**Figure 5:**
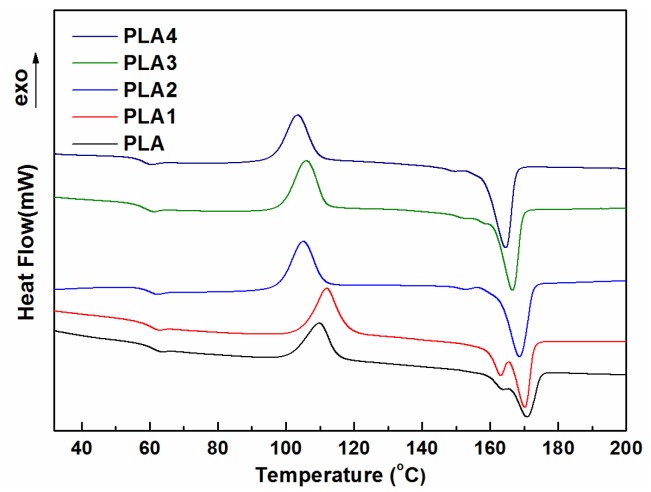
DSC curves of PLA and PLA/DAP blends.

DSC was adopted to study the effect of DAP on the glass transition temperature (*T*_g_), crystallization, and melting temperatures (*T*_m_) of PLA, and the corresponding parameters are illustrated in Figure 5 and Table 4. PLA shows a *T*_g_ value at 61.0 °C, an exothermic cold crystallization peak at 109.8 °C, and an endothermic peak at 170.4 °C, with a shoulder peak at lower temperature. This phenomenon of PLA is reported to be a result of lamellar rearrangement during crystallization of PLA, as well as the reorganization of poor crystalline regions with different crystalline structures within PLA [34,35]. It is observed that after the addition of DAP to PLA, *T*_g_ values of PLA1, PLA2, PLA3, and PLA4 gradually decrease from 61.0 °C of PLA to 60.3, 59.4, 58.2, and 58.0 °C, owing to the enhanced molecular chain mobility. With regard to the *T*_c_ values of PLA/DAP blends, they, on the whole, decrease to some extent, and the crystallization ability of PLA is improved due to the increased segmental mobility of PLA chains by plasticization of DAP. Furthermore, the *T*_m_ values of them also decrease considerably. Meanwhile, it is noted that the shoulder peaks of PLA/blends disappear, indicating the formation of crystallite with same sizes and perfection during crystallization of PLA. Through calculation, it confirms that DAP actually increases the degree of crystallinity (*X_c_*) of PLA, and *X*_c_ gradually increases with the increasing loading of DAP, which verifies the abovementioned analyses.

### 3.4. Flame-Retardant Mechanism

**Figure 6:**
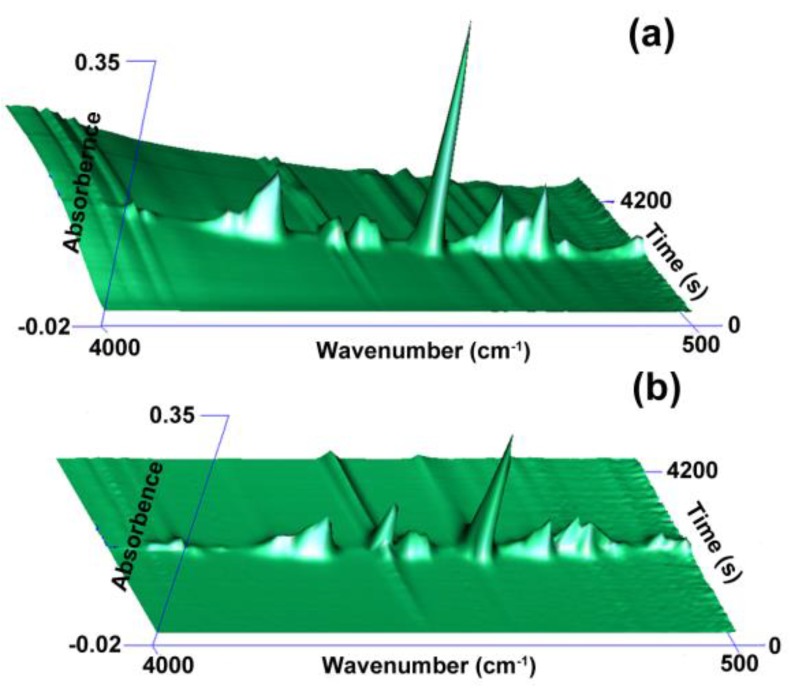
The 3D-surface FTIR spectra of volatiles of PLA (**a**) and PLA4 (**b**).

It is known that the removal of melt dripping without flame can bring away heat and even fuels produced by the decomposition of PLA [36]. However, what is the key to the production of melt dripping without flame? According to the results of TG and CC tests, it indicates that DAP works mainly in the gaseous phase since the char residues are improved so slightly that they cannot be considered as a barrier to insulate the transfer of heat and oxygen. Therefore, in order to understand the gas-phase actions of DAP, the evolved gas products of PLA and PLA4 were analyzed by TG-FTIR. From the two 3D-surface FTIR spectra as seen in Figure 6, the majority peaks related to the gaseous products are same, while the difference exists in the emerging temperature and intensity of peaks.

**Figure 7:**
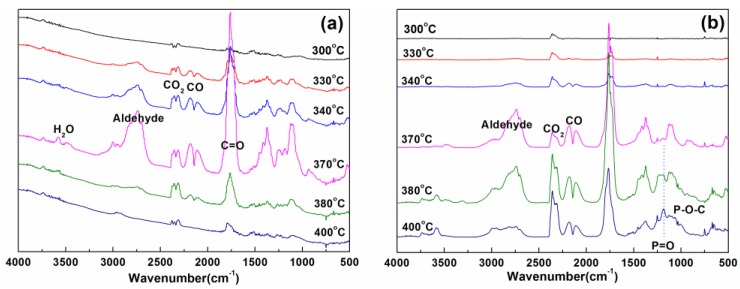
FTIR spectra of volatile products for PLA (**a**) and PLA4 (**b**) at different temperatures.

To make the difference between the volatiles of PLA and PLA4 clear, FTIR spectra of the volatiles selected at variable temperatures are depicted in Figure 7. It is reasonable to recognize the products according to the characteristic peaks that PLA decomposes to produce H_2_O, hydrocarbons, aldehyde, CO_2_, CO, and carbonyl compounds, such as lactide, which corresponds to the previous reports [17,18,37]. By comparing the two spectra of PLA and PLA4, it is found that DAP has little effect on changing the decomposition products of PLA, while the main difference is noted at 380 and 400 °C, that water, hydrocarbons, and aldehyde are still produced until 400 °C for PLA4. In addition, through comparing the intensity of CO_2_/CO for PLA and PLA4, it can be found that PLA4 releases more CO_2_. It is known that the CO_2_ production is mainly derived from the decarboxylic reaction of lactide, that is, the addition of DAP might promote the production of lactide. Besides, the absorption peaks of P=O at about 1250 cm^−1^ and P–O–C at about 1105 cm^−1^ are found in the spectrum of PLA4, proving that DAP actually discharges phosphorus-containing compounds to the vapor phase.

**Figure 8:**
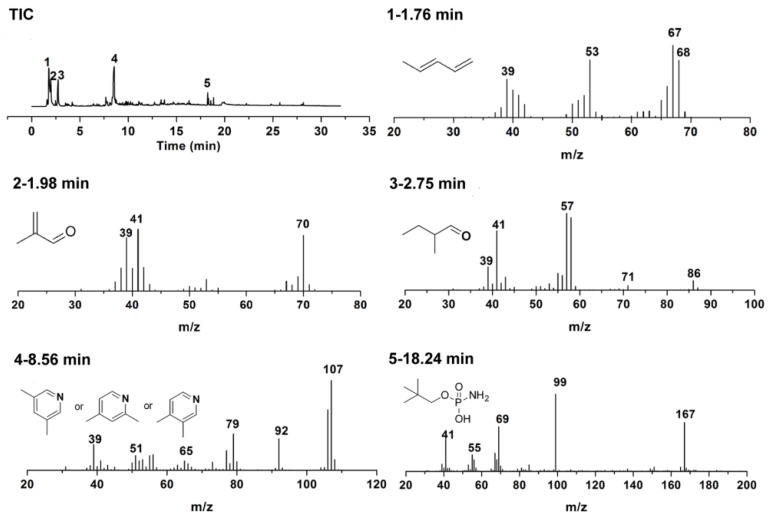
The TIC and HR-MS spectra of the pyrolysis products of DAP.

Py-GC/MS is now widely used and very effective to detect the pyrolysis products of materials [38,39,40]. In order to further identify the release of phosphorus-containing compounds of DAP in the gaseous phase, py-GC/MS was adopted, and the relevant total ion chromatogram (TIC) and high-resolution mass spectra (HR-MS) are illustrated in Figure 8. From the TIC spectrum, it is found that the pyrolysis products of DAP are very complicated, so the main products of DAP detected at 1.76, 1.98, 2.75, 8.56, and 18.24 min are selected, including 1,3-pentadiene, methacrolein, 2-methylbutyraldehyde, dimethylpyridine, and phosphorus-containing derivative of DAP. Based on the chemical structure of DAP, it is speculated that methacrolein and 2-methylbutyraldehyde might be produced from the pyrolysis of AMPD, while the detected phosphorus-containing derivative might be generated by the multi-reaction of intermediates. All in all, phosphorus-containing fragments are actually detected, which is in accordance with the results of TG-FTIR.

In conclusion, DAP plays a super-effective role in flame-retarding PLA through gaseous activities, including the production of non-flammable gases, such as water and ammonium, with the ability of diluting the oxygen and fuel concentration, and release of phosphorus-containing fragments that can produce phosphorus-containing free radicals with the function of interrupting free-radical reactions during combustion. Additionally, it is certain that the removal of non-flammable melt dripping also helps to bring away heat and cut off the heat-transfer to the polymer.

## 4. Conclusions

These studies developed a novel flame retardant, namely dihydroxy-containing ammonium phosphate (DAP), and its chemical structure was well characterized and confirmed. It showed that DAP was fit to flame-retard PLA with a relative low loading, and accordingly, PLA/DAP materials maintained good thermal stability as compared to PLA, that the *T*_5%_ and *T*_max_ values of PLA/DAP blends were still close to that of PLA. In the presence of DAP, particularly when the loading of DAP was only 0.5 wt %, the LOI value and UL-94 rating reached 24.6% and V-0 rating. The improved flame retardancy of PLA was because of the flame-inhibition activities of DAP in the gaseous phase, including the production of non-flammable gases, such as water and ammonium, and the release of phosphorus-containing fragments. Based on this, it led to the production of noncombustible melt dripping, which could bring away a large amount of heat and stop the feedback of heat to the matrix. While influenced by plasticization of DAP, the chain mobility of PLA was enhanced, resulting in that the glass transition temperatures and cold crystallization temperatures of PLA/DAP blends all decreased, while the degree of crystallinity increased. All in all, it was concluded that DAP showed high efficiency on flame-retarding PLA, while not reducing the thermal stability of PLA.

## Figures and Tables

**Table 1:** The formulation and fire-testing results for poly(lactic acid) (PLA) and PLA/DAP blends.

Samples	PLA (wt %)	DAP (wt %)	LOI (%)	UL-94 (3.2 mm)	Dripping	Igniting Cotton
PLA	100	0	19.5	NR	Yes	Yes
PLA1	99.75	0.25	23.0	V-2	Yes	Yes
PLA2	99.5	0.5	24.6	V-0	Yes	No
PLA3	99	1	30.3	V-0	Yes	No
PLA4	98	2	38.3	V-0	Yes	No

**Table 2:** The detailed data derived from cone calorimeter (CC) tests for PLA and PLA/DAP blends.

Samples	TTI (s)	PHRR (kW m^−2^)	THR (MJ m^−2^)	TTP (s)	FIGRA (kW m^−1^ s^−1^)	TSP (m^3^)	av-EHC (MJ/kg)
PLA	54 ± 1	436 ± 25	64.2 ± 0.7	170 ± 10	2.56	24.6 ± 1.5	17.5 ± 0.5
PLA3	71 ± 2	439 ± 10	62.8 ± 1.5	175 ± 10	2.50	24.6 ± 2.0	16.7 ± 0.5
PLA4	69 ± 1	463 ± 18	60.8 ± 1.0	190 ± 15	2.44	25.1 ± 2.5	15.6 ± 0.9

**Table 3:** Thermal parameters derived from TG for DAP, PLA, and PLA/DAP blends.

Samples	*T*_5%_ (°C)	*T*_max_ (°C)	Mass Loss Rate at *T*_max_ (wt %·min^−^^1^)	Residual at 600 °C (wt %)
P	237	306	21.7	17.1
PLA	335	368	32.5	2.3
PLA1	333	370	31.7	2.6
PLA2	335	370	32.5	3.1
PLA3	335	370	32.0	3.5
PLA4	329	370	31.3	3.5

**Table 4:** DSC parameters of PLA and PLA/DAP blends.

Samples	*T*_g_ (°C)	*T*_c_ (°C)	Δ*H*_c_ (J g^−1^)	*T*_m_ (°C)	Δ*H*_m_ (J g^−1^)	*X*_c_ (%)
PLA	61.0	109.8	28.48	170.4	31.55	3.3
PLA1	60.3	112.0	34.03	169.8	38.39	4.7
PLA2	59.4	105.1	31.03	168.4	39.27	8.9
PLA3	58.2	106.1	33.10	166.1	42.28	10.0
PLA4	58.0	103.4	30.08	164.1	41.99	12.3
